# rdHSV-CA8 non-opioid analgesic gene therapy decreases somatosensory neuronal excitability by activating Kv7 voltage-gated potassium channels

**DOI:** 10.3389/fnmol.2024.1398839

**Published:** 2024-05-09

**Authors:** Munal B. Kandel, Gerald Z. Zhuang, William F. Goins, Marco Marzulli, Mingdi Zhang, Joseph C. Glorioso, Yuan Kang, Alexandra E. Levitt, Wai-Meng Kwok, Roy C. Levitt, Konstantinos D. Sarantopoulos

**Affiliations:** ^1^Department of Anesthesiology, Perioperative Medicine and Pain Management, University of Miami Miller School of Medicine, Miami, FL, United States; ^2^Department of Microbiology and Molecular Genetics, University of Pittsburgh School of Medicine, Pittsburgh, PA, United States; ^3^Bascom Palmer Eye Institute, University of Miami Miller School of Medicine, Miami, FL, United States; ^4^Department of Anesthesiology and Department of Pharmacology & Toxicology, Medical College of Wisconsin, Milwaukee, WI, United States; ^5^John T. MacDonald Foundation Department of Human Genetics, University of Miami Miller School of Medicine, Miami, FL, United States; ^6^John P. Hussman Institute for Human Genomics, University of Miami Miller School of Medicine, Miami, FL, United States

**Keywords:** non-opioid analgesia from CA8 gene therapy, carbonic anhydrase-8, Kv7 voltage-gated potassium channels, neuronal excitability, afterhyperpolarization, replication defective herpes-1 virus, gene therapy

## Abstract

Chronic pain is common and inadequately treated, making the development of safe and effective analgesics a high priority. Our previous data indicate that carbonic anhydrase-8 (CA8) expression in dorsal root ganglia (DRG) mediates analgesia via inhibition of neuronal ER inositol trisphosphate receptor-1 (ITPR1) via subsequent decrease in ER calcium release and reduction of cytoplasmic free calcium, essential to the regulation of neuronal excitability. This study tested the hypothesis that novel JDNI8 replication-defective herpes simplex-1 viral vectors (rdHSV) carrying a CA8 transgene (vHCA8) reduce primary afferent neuronal excitability. Whole-cell current clamp recordings in small DRG neurons showed that vHCA8 transduction caused prolongation of their afterhyperpolarization (AHP), an essential regulator of neuronal excitability. This AHP prolongation was completely reversed by the specific Kv7 channel inhibitor XE-991. Voltage clamp recordings indicate an effect via Kv7 channels in vHCA8-infected small DRG neurons. These data demonstrate for the first time that vHCA8 produces Kv7 channel activation, which decreases neuronal excitability in nociceptors. This suppression of excitability may translate *in vivo* as non-opioid dependent behavioral- or clinical analgesia, if proven behaviorally and clinically.

## Introduction

Chronic pain is common and inadequately treated, making the development of safe and effective analgesics a high priority. Our previous studies unraveled a novel analgesic pathway involving carbonic anhydrase-8 (CA8) expression in primary afferent neurons. CA8 mediates analgesia (Fu et al., 2017; Levitt et al., 2017; Upadhyay et al., 2019) via inhibition of neuronal ER inositol trisphosphate receptor-1 (ITPR1), a subsequent decrease in ER calcium release, and a reduction of cytoplasmic free calcium, essential to the regulation of neuronal excitability (Berridge, 1993; Hirasawa et al., 2007; Zhuang et al., 2015; Fu et al., 2017; Levitt et al., 2017; Zhuang et al., 2018; Upadhyay et al., 2019). This novel analgesic pathway has the potential to address the unmet need for effective new non-opioid analgesics to treat chronic pain conditions [Institute of Medicine (US) Committee on Advancing Pain Research, Care, and Education, 2011]. The impact of chronic pain disorders is enormous and costly (McNicol et al., 2013; Busse et al., 2018). In the absence of suitable analgesic alternatives to treat chronic noncancer pain, an epidemic of opioid overuse, abuse, and life-threatening complications has occurred (Compton and Volkow, 2006; Paulozzi and Xi, 2008; Boudreau et al., 2009; Edlund et al., 2010a,b; Ostling et al., 2018).

To address the hypothesis that V5-CA8 (modified human CA8) represents a novel non-opioid analgesic, we have previously delivered V5-CA8 to DRG via sciatic nerve injection in mice using adeno-associated virus-based (AAV) gene therapy. This AAV-V5-CA8 gene therapy vector transduced DRG of mice to produce profound, long-lasting analgesia (equivalent >100 mg of oral morphine in 60 kg adult for more than 4 weeks) and treated chronic pain in various models (Berridge, 1993; Hirasawa et al., 2007; Zhuang et al., 2015, 2018; Upadhyay et al., 2019, 2020). Unlike local anesthetics, V5-CA8-related analgesia occurred without motor blockade or clinical pathology (Berridge, 1993; Hirasawa et al., 2007; Zhuang et al., 2015, 2018; Fu et al., 2017; Levitt et al., 2017; Upadhyay et al., 2019, 2020). Yet, despite the fundamental role of CA8 in regulating intracellular calcium signaling, our understanding of how CA8 regulates neuronal excitability to produce analgesia remains unknown.

One potential mechanism is via the opening of Kv7 voltage-gated potassium channels. K_V_7 channels are the only known neuronal potassium channels activated by lower cytoplasmic calcium to produce M-currents (I_M_) through calmodulin-dependent and -independent mechanisms (Selyanko and Brown, 1996a; Gamper and Shapiro, 2003; Delmas and Brown, 2005; Burgoyne, 2007; Kosenko and Hoshi, 2013). I_M_ regulates neuronal excitability and produces analgesia by prolonging neuronal AHP, which restricts the firing of action potentials and the propagation of afferent nociceptive signals (Lombardo and Harrington, 2016; Wu et al., 2017). Kv7 voltage-gated potassium channel openers (e.g., flupirtine, retigabine) are well-known to produce non-opioid-based analgesia in various animal models and human chronic pain conditions (McMahon et al., 1987; Mastronardi et al., 1988; Blackburn-Munro and Jensen, 2003; Erichsen et al., 2003; Passmore et al., 2003; Munro and Dalby-Brown, 2007; Wulff et al., 2009; Perdigoto et al., 2018). However, despite their utility in treating chronic pain, all previous Kv7 channel openers were removed from the market due to adverse events after their oral use and systemic exposure (Konishi et al., 2018; Perdigoto et al., 2018). Nonetheless, Kv7 channels remain important analgesic targets. Based on our findings, we speculate that the activation of Kv7 channels by CA8-mediated reduction of cytoplasmic free calcium explains the analgesia observed through prolonged AHP and reduced neuronal excitability.

A significant limitation of AAV strains used in our prior studies was their limited potential to transduce DRG neurons except after direct intra-neural injections (Chan et al., 2017; Chakrabarti et al., 2020). We used rdHSV vectors to transduce neuronal cultures in the current study to address this limitation. rdHSV-based gene therapy has the potential to transduce DRG neurons after intra-articular, intradermal, and intra-neural injections commonly used in chronic pain treatments. JDNI8 HSV gene therapy vectors are replication-defective and disease-free due to the deletion of all the viral immediate early (IE) genes (Miyagawa et al., 2015, 2017; Verlengia et al., 2017). These replication defective (rd)HSV vectors provide an efficient delivery system to the peripheral nervous system that selectively establishes natural lifelong latency within the nucleus of infected neurons, following retrograde transport of viral particles to the nerve cell bodies in DRG. Together, these viral modifications provide a non-cytotoxic vector capable of long-term episomal maintenance in neurons with at least 6 months of continued, robust transgene expression (Verlengia et al., 2017). These vectors are non-cytotoxic, provide no viral antigen targets for immune effector cells, and are consequently much less likely to produce local inflammation.

The primary goal of this study was to apply this novel gene therapy system to test the hypothesis that expression of a human V5-CA8 peptide variant in small primary afferents, likely nociceptors, can attenuate their excitability, via a mechanism that involves CA8-induced prolongation of their afterhyperpolarization (AHP) resulting from the activation of their Kv7 voltage-gated potassium channels. The specificity of the treatment was confirmed using a null-mutant CA8 gene vector and *in vitro* drug-mediated (XE-991) selective antagonism of the Kv7 channels.

## Materials and methods

### Animal preparations and care

All procedures related to animal use and care were preapproved by the University of Miami Institutional Animal Use and Care Committee (IACUC). Sprague Dawley (SD) rats used in DRG neuronal culture experiments were 1–2 weeks of age. All animals were housed in a facility at a controlled temperature and humidity. A 12 h light/dark cycle and water and food *ad libitum* were provided.

### Engineering of vHCA8WT and vHCA8MT viruses

The WT and MT vHCA8 HSV vectors were made by simply digesting the HCA8-V5-AAV-MCS4650 plasmids both wildtype (WT) and mutant (MT) (Zhuang et al., 2018) with BglII enzyme (New England Biolabs) (Upadhyay et al., 2020). Gibson Reaction (NEBuilder HiFi DNA Assembly, New England Biolabs, Ipswich, MA) was used to clone those BglII fragments upstream of a PCR fragment of T2A-GFP sequence from a glycoprotein C (gC)-T2A-eGFP fusion plasmid (Mazzacurati et al., 2015) using the following primers: T2A-GFP-F: 5-'CTCGGTCTCGATTCTACGGAGGGCAGAG-GAAGTCTGCTAACATGCGGTGACGTCGAGGAGAATCCTGGCCCAGAGAGCGACGAGAGCGGCCT-3′, GFP-R: 5’-AGGGATGCCACCCGTAGATCT-tta-GCGAGATCCG-GTGGAGCCGG-3′. The final products from the Gibson Reaction, pAAV-CAGp-hsCA8(WT)-V5-T2A-GFP and pAAV-CAGp-hsCA8(MT)-V5-T2A-GFP were then transferred as 3473-bp NotI-digested gel isolated bands into the NotI site of ccdB^−^ pENTER 1A between the attL recombination sites to create the final products named pE-CAGp-hsCA8(WT)-V5-T2A-GFP and pE-CAGp-hsCA8(MT)-V5-T2A-GFP (Mazzacurati et al., 2015; Miyagawa et al., 2015). The LR gateway reaction using LR Clonase (ThermoFisher, Pittsburgh, PA) was used to insert the cassettes from these plasmids into JDNI8-GW41 BAC vector purified from HH8 bacteria (Miyagawa et al., 2015). Recombinants were screened by PCR across the GW cassette and confirmed by field inversion gel electrophoresis (FIGE) analysis (FIGE mapper, BioRad, Hercules, CA) of restriction enzyme digests of the recombinants (LaBranche et al., 2017).

### Preparation, purification, and authentication of HSV-V5-CA8 virus particles

The JDNI8-CAGp-V5-CA8WT-T2A-GFP (vHCA8WT) or JDNI8-CAGp-V5-CA8MT-T2A-GFP (vHCA8MT) vectors were produced by transfection of U2OS-4/27 complementing cells with DNA purified from BAC preps for each of the vectors. Individual isolates were purified using limiting dilution analysis, and then small virus stocks were used to infect Cre-expressing ICP4/ICP27-complementing (U2OS-4/27-Cre) cells to eliminate the BAC sequences by Cre-mediated recombination (LaBranche et al., 2017; Goins et al., 2020). Limiting dilution analyses were again performed, and individual isolates lacking the BAC were identified by X-gal staining of individual plaques in 96-well plates (Thermo-Fisher, Pittsburgh, PA). Following BAC deletion, viral stocks were grown to high titer (Ozuer et al., 2002; Wechuck et al., 2002; Goins et al., 2014, 2020) and used to infect 1× 10-layer Cell Factory (Corning, Corning, NY) of U2OS-4/27 complementing cells at MOI = 0.0005 in VP-SFM MEDIA (Thermo-Fisher, Pittsburgh, PA) for 1-h at 37°C in a CO_2_ incubator. On ~day 8, the CFs displayed ~90% CPE, and the next day, NaCl was added to 0.45 M, and the CFs rocked for 4-h. Virus supernatant was harvested and processed by 0.8-micron CN filtration (Thermo-Fisher, Pittsburgh, PA) and subjected to centrifugation at 43,000xg for 45–90 min, followed by a dPBS wash and a second identical centrifugation step. The vector was finally resuspended in dPBS with sterile glycerol added to a final volume of 10%, and the virus was vialed in 10 μl (actual volume 12.5 μl) aliquots in cryovials and stored at –80°C. The overall titers were determined by standard plaque assay on U2OS-4/27 complementing cells (Goins et al., 2020). Aliquots of 10 μl were used for QA/QC testing and expression/toxicity in primary rat DRGs. Toxicity was assessed by MTT assay that showed the vHCA8WT vector was like the vHCA8MT control vector on primary rat DRG or mock-infected DRG.

### DRG neuronal primary culture

DRG were dissected from 1 to 2 weeks old Sprague Dawley (SD) rats anesthetized with isoflurane and decapitated under deep isoflurane anesthesia. DRG were harvested and then enzymatically digested with 5 mg/mL collagenase/Dispase (Roche) for 2 h at 37°, followed by 0.25% trypsin (Gibco) for 10 min at 37°C. The enzymatic reaction was stopped by adding 0.25% trypsin inhibitor and DMEM containing 10% fetal bovine serum and 1% penicillin and streptomycin. The ganglia were then mechanically triturated with a fire-polished Pasteur pipette of three different sizes and passed through a 40-μM cell strainer. The dissociated neurons were then centrifuged and resuspended in complete neurobasal medium [neurobasal medium (Gibco) containing 1% penicillin and streptomycin (Gibco), 1% L-glutamine (Gibco), 2% B27 supplement (Gibco), 100 ng/mL nerve growth factor].

For electrophysiological (EP) recording, dissociated neuronal somata were plated on 15 mm glass coverslip (Electron Microscopy Sciences, PA) coated with Poly-D-Lysine/laminin at a density of 2 × 104 cells/well. Cells were maintained at 37°C in a water-saturated atmosphere containing 5% CO_2_ and 95% air. Cultured DRG neurons were transduced with 2 × 10^4^ PFU of vHCA8WT or vHCA8MT vectors or remained non-infected as a negative control.

### Whole-cell patch-clamp recordings

**Figure 1:**
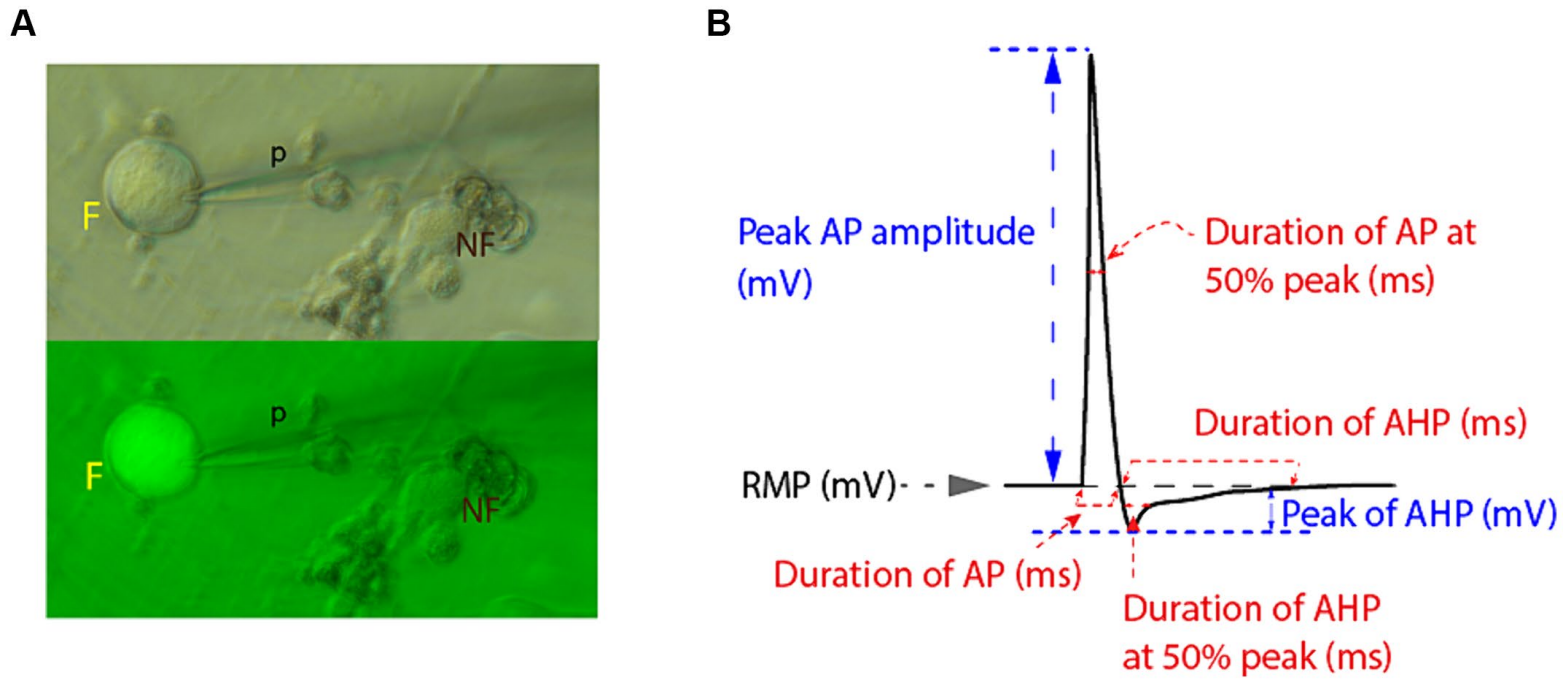
Electrophysiologic recordings and measurements from small diameter DRG neuronal somata. **(A)** Small diameter DRG neuronal somata were selected for patch clamping by size ≤30 μm in bright field microscopy and by active fluorescent status (F) under green fluorescence microscopy (p, recording micropipette; NF, non-fluorescent cell). **(B)** Schematic representation of measurements of relevant electrophysiological parameters of recorded AP and of AHP. Measurements were obtained using Clampfit software.

EP experiments were performed 48–72 h after transduction. Neuronal somata were viewed using Hoffman modulation optics under a Nikon Eclipse Ti inverted microscope, and then only small diameter (<30 μm) neurons (as determined by microscope eyepiece reticle), selected by GFP fluorescence, were further studied (Figure 1A). These small-diameter (<30 μm) neurons correlate with electrophysiological characteristics corresponding to nociceptive C-fibers (Lawson, 2002).

Recordings were obtained using the whole-cell configuration of the patch-clamp technique in current-clamp mode, wherein APs were elicited by brief depolarizing square step current commands in external Tyrode’s solution at room temperature. Recordings were conducted via an Axon Multiclamp 700B amplifier and an Axon 1550A digitizer and analyzed using Clampfit. Initially, resting membrane potential (RMP) was recorded at baseline, and neurons with RMP more depolarized than −45 mV, indicating a large leak current, were rejected. Neurons with unstable or distorted recordings or excess “noise” were also rejected. The firing of AP was evoked in response to a sequence of five 3 ms square current commands, repeated every 2000 ms, and increasing from 500 to 2500 pA (i.e., 1st step 500 pA, 2nd step 1000 pA, 3rd step 1500 pA, 4th step 2000 pA and 5th step 2500 pA). Monomorphic AP and their AHPs were captured in subsequent recordings lasting 2000 ms (to measure both AP and longer-lasting AHP parameters), and the APs elicited by the fifth current command (2500pA) were used for further analysis. Parameters of AP were measured in Clampfit and compared between groups. AP duration was measured at baseline (at the beginning of the sharp upward rise of the depolarizing phase until returning to baseline) as well as at 50% amplitude (from the point where a horizontal diachronic line was drawn from the rising phase at 50% amplitude to the point where the descending, repolarizing phase crosses this line). AP amplitude was measured from RMP to the AP peak, while AHP amplitude was measured from the RMP to the most hyperpolarized (negative) level of the AHP phase. AHP duration was measured at points representing 50 and 100% recovery back to RMP (see Figure 1B). Figure 1B provides a schematic representation of relevant EP parameters measured, including those of resting membrane potential (RMP), of action potential (AP) and of AHP phase.

**Figure 2:**
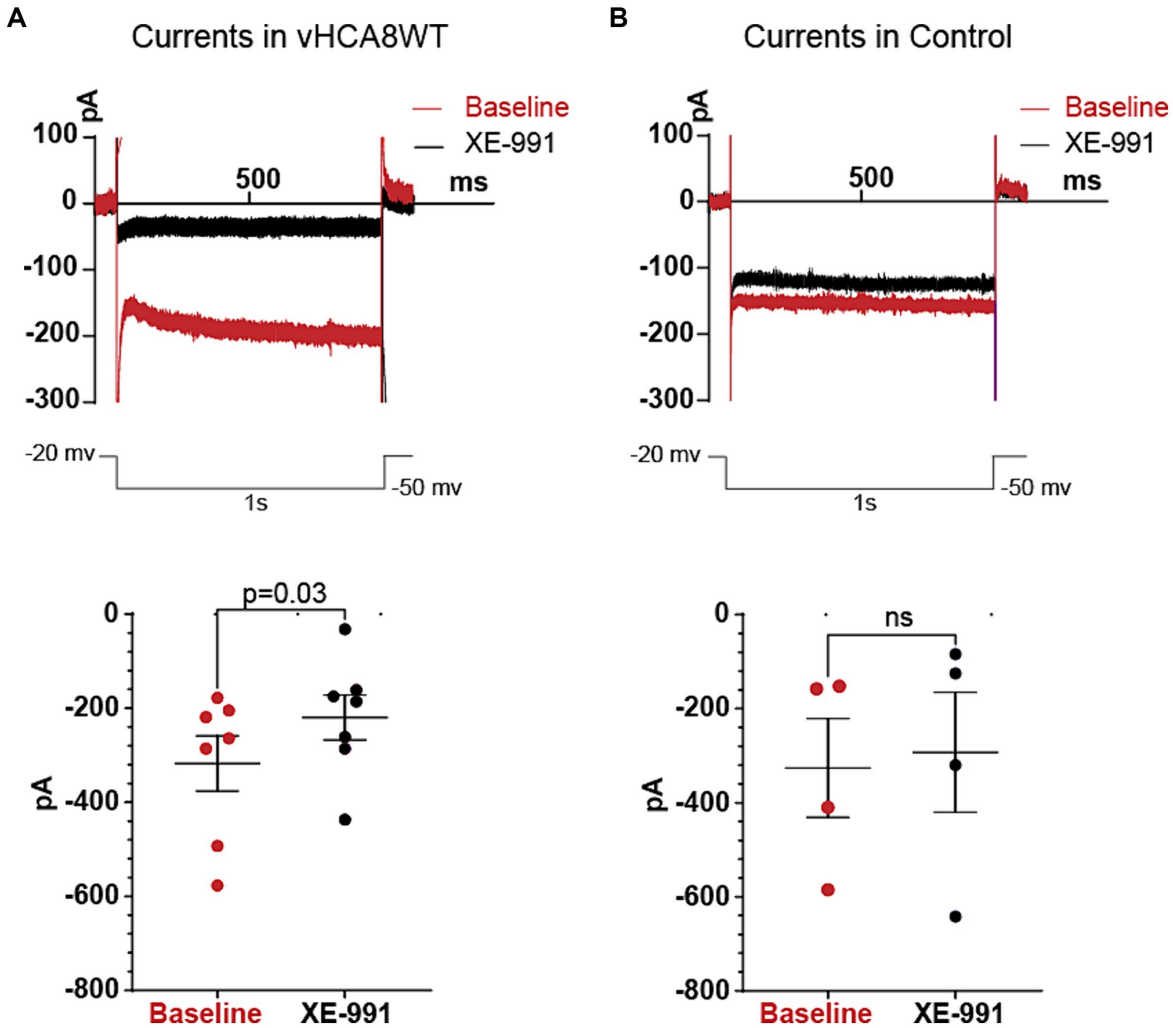
Differences in Kv7 currents between vHCA8WT DRG neuronal somata versus Controls. Current responses from −20 mV holding potentials to −50 mV hyperpolarizing voltage command steps of 1 s duration were recorded before and after administration of 10 μM XE-991, in vHCA8WT-infected and control (non infected) neurons. The upper two traces in Figure 2 show representative original traces from a vHCA8WT transduced neuron in the left panel **(A)** and from a Control (non-infected) neuron in the right panel **(B)**. The lower two plots show the measured values in each individual neuron tested, with each specific dot representing a current value recorded from each neuron, at baseline (red) and after XE-991 (black). Again, the left sided plot **(A)** indicates the changes in vHCA8WT treated neurons and the right sided plot **(B)** refers to non-infected control neurons. Mean values are shown in the longer horizontal bar and SEM of these groups at the shorter horizontal bars. The selective Kv7 inhibitor XE-991 had an inhibitory effect only in vHCA8WT infected cells, indicating the presence of significant Kv7 currents only in vHCA8WT infected cells.

For voltage-clamp recordings, neurons were first patched in a current-clamped mode. After switching to voltage-clamp, currents in DRG cells were recorded by holding the membrane potential at −20 mV and applying a square-form hyperpolarizing pulse to −50 mV for a duration of 1 s, and then back to –20 mV (Figure 2).

Patch pipettes were pulled from borosilicate glass capillaries (Sutter instrument O.D.:1.5 mm, I.D.:0.86 mm) using Sutter Instrument P-87 Flaming/Brown Micropipette Puller and flame polished with a micro forge polisher (Narishige, Japan) with a tip resistance of 4–8 MΩ when filled with internal solution.

For current-clamp recording, pipettes were filled with an internal solution containing (in mM) 120 KCl, 5 Na-ATP, 0.4 Na-GTP, 5 EGTA, 2.25 CaCl_2_, 5 MgCl_2_, and 20 HEPES (adjusted to pH 7.4 with KOH). The external Tyrode’s solution contained (in mM): 140 NaCl, 4 KCl, 2 CaCl_2_, 10 glucose, 2 MgCl_2_, and 10 HEPES (adjusted to pH 7.4 with NaOH).

For voltage-clamp recording, pipettes were filled with an internal solution containing (in mM): 80 K acetate, 30 KCl, 40 HEPES, 3 MgCl_2_, 3 EGTA and 1 CaCl_2_ (adjusted to pH 7.4 with NaOH). The external solution contained (in mM): 144 NaCl, 2.5 KCl, 2 CaCl_2_, 10 glucose, 0.5 MgCl_2_, 5 HEPES (adjusted to pH 7.4 with Tris base). Gravity-driven whole bath perfusion was used in all recordings.

The Kv7 blocker XE-991 was prepared in the external solution at a final concentration of 10 μM and perfused in the bath via the same gravity-driven perfusion.

Only fluorescent small-sized DRG neurons with diameter ≤ 30 μM, as determined by microscope eyepiece reticle, were selected for the electrophysiological recordings.

### Reagents

All chemicals or reagents were obtained from and authenticated by Sigma-Aldrich, St. Louis, MO, except as otherwise mentioned in the text.

### Statistical analysis

Statistical analyses were conducted with unpaired and paired Student’s *t*-tests.

The GraphPad Prism software was used for statistical analyses and plotting graphs. Adobe Illustrator was then used to format the figures further.

## Results

### Expression of WT and MT HCA8 in cultured primary DRG neurons after transduction with vHCA8 vectors

For these studies, we used JDNI8-CAGp-V5-CA8WT-T2A-GFP (vHCA8WT) and JDNI8-CAGp-V5-CA8MT-T2A-GFP (vHCA8MT) constructs containing the wildtype CA8-201 (WT) and CA8 mutant (MT)(CA8 cDNAs containing the S100P null point mutation) (Turkmen et al., 2009) modified with a V5 tag. These transgenes were inserted downstream of the CAG promoter into these later-generation nontoxic replication-defective vectors at the ICP4 site (Supplementary Figure S1A; Miyagawa et al., 2015). The CA8MT (CA8 S100P) represents a rigorous negative control due to the nearly complete loss of CA8 cellular protein associated with rapid proteasome-mediated degradation (Turkmen et al., 2009). We used a strong promoter system based on the cytomegalovirus early enhancer element; the promoter region, first exon and the first intron of the chicken beta-Actin gene; and the splice acceptor of the rabbit beta-Globin gene (CAG) that is known to be active in a broad array of cell types including neurons to evaluate the possibility that expression of CA8 in additional cell types may occur and contribute to the metabolic control of the analgesic response (Zhuang et al., 2018). These viral particles were used to transduce cultured rat primary DRG neurons (Supplementary Figure S1B). To verify that the vectors produce the proper sized CA8 protein product in DRG neurons in culture, vector-infected DRG cell lysates harvested 2 days post-infection were employed in western blot analyses using the CA8 and V5 tag antibodies (Supplementary Figure S1B). We demonstrated that the vHCA8WT vector yielded high levels of V5-CA8 product detected with both V5 and CA8 antibodies while the vHCA8MT vector expressed greatly reduced levels of the CA8 protein in comparison to the ß-actin loading control, similar to that seen previously with the AAV-CA8WT and MT vectors. We showed that the eGFP reporter gene by IHC was co-expressed from the same CAG promoter-driven message that encodes vHCA8WT/MT due to the use of a T2A self-cleaving site (Szymczak and Vignali, 2005) almost exclusively in neurons using the pan-neuronal advillin (AVIL) antibody and in specifically in Nav1.8-specific C-fiber neurons. These *in vitro* studies confirmed that our vectors produce high levels of the correct-sized WT gene product in neuronal cells transduced by the HSV replication-defective vectors.

### vHCA8 prolongs AHP in primary afferent neurons

**Figure 3:**
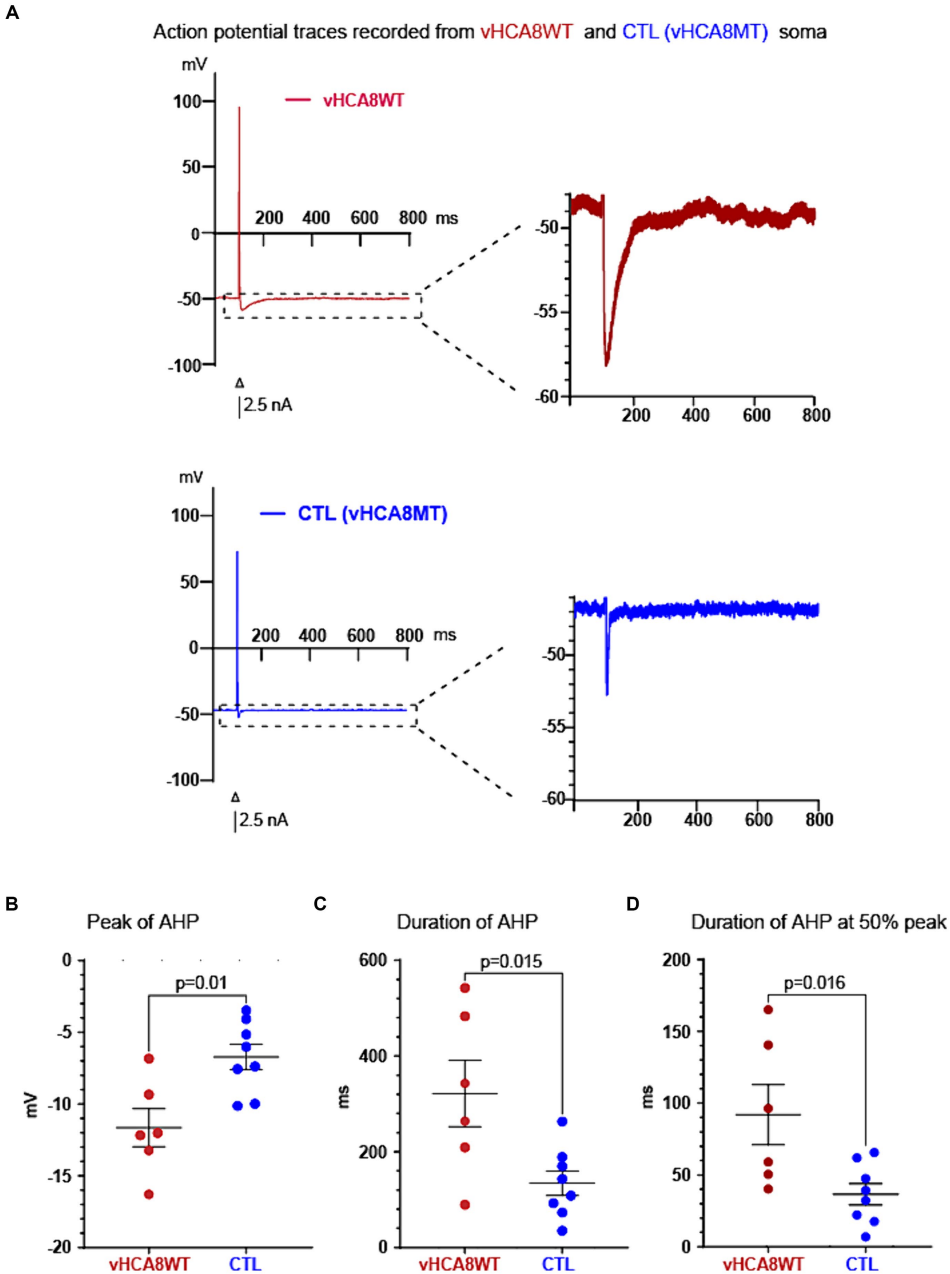
vHCA8WT prolongs AHP in infected DRG neuronal somata. **(A)** Representative AP traces recorded from vHCA8WT (red) and from vHCA8MT (blue) infected neuronal somata are shown. AP firing was elicited by brief depolarizing current command steps of 2.5 nA amplitude (shown as black bar under the action potentials). The apparent differences in the peak AHP amplitude and in the duration of the AHP are shown. **(B)** Impact of vHCA8WT infection on AHP dimensions. DRG neuronal somata after infection with vHCA8WT (red circles) versus controls (including vHCA8MT and uninfected cells) (blue circles) show a more negative peak AHP amplitude. Similarly, DRG neuronal somata after infection with vHCA8WT (red circles) versus controls (including vHCA8MT and non-infected cells) (blue circles) show prolonged AHP duration at baseline level - corresponding to the resting membrane potential level **(C)**, and at the level of the 50% of peak AHP amplitude **(D)**. Means ± SEM are shown. Horizontal bars above groups indicate statistically significant differences between groups.

To test the hypothesis that vHCA8 infection reduces neuronal excitability, we obtained whole-cell current clamp recordings from isolated small-sized (≤ 30 microns) DRG neuronal somata (Figure 3 and Table 1) infected with vHCA8WT and controls (vHCA8MT and non-infected). Small diameter vHCA8 infected neurons (vHCA8WT) were selected under bright field and green fluorescence microscopy (Figure 1A) to detect GFP expression since eGFP is linked to HCA8 via the T2A site.

**Table 1:** Comparison of electrophysiological parameters between small-sized DRG neuronal somata infected with vHCA8WT versus controls (infected with vHCA8MT or uninfected).

	**vHCA8WT (*n* = 6)**	**Controls (*n* = 8)**	***p*-value**
Soma diameter (μ)	23.67 ± 3.82	24.25 ± 1.9	0.7132
Resting membrane potential (mV)	−51.54 ± 4.61	−50.23 ± 4.55	0.6090
Peak action potential amplitude (mV)	141.95 ± 26.62	147.02 ± 24.63	0.7227
Duration of action potential (ms)	9.58 ± 3.53	8.75 ± 3.24	0.6603
Duration of action potential at 50% amplitude (ms)	3.85 ± 1.63	3.12 ± 0.73	0.2842
Peak afterhyperpolarization amplitude (mV)	−11.65 ± 3.25	−6.72 ± 2.5	0.0075
Duration of afterhyperpolarization (ms)	321.77 ± 170.45	134.24 ± 72	0.0155
Duration of afterhyperpolarization at 50% AHP peak amplitude (ms)	91.93 ± 51.39	36.63 ± 20.96	0.0167

Data are shown for vHCA8WT-infected DRG cultured somata versus controls (infected with vHCA8MT or uninfected cells). No differences were detected between these two latter groups (data not shown); therefore, both subgroups were combined and served as a single control group. Means ± SDs are shown. Comparisons were carried out with unpaired Student’s *t*-tests (testing the hypothesis that vHCA8WT reduces excitability parameters).

Neuronal somata infected with vHCA8WT and controls did not differ in size, RMP, AP peak amplitude, and AP duration (Table 1). However, neuronal somata infected with vHCA8WT exhibited much larger AHP. Representative traces are shown in Figure 3A. Specifically, vHCA8WT AHPs, when compared to controls, exhibited significantly larger AHP peak amplitude with −11.65 ± 3.25 vs. −6.72 ± 2.5 mV (*p* < 0.01), longer AHP duration 321.77 ± 170.45 vs. 134.24 ± 72 msec (*p* < 0.05) and longer AHP duration at 50% amplitude 91.93 ± 51.39 vs. 36.63 ± 20.96 msec (*p* < 0.05), respectively (Table 1 and Figures 3B–D).

Because prolonged AHP results in decreased neuronal excitability, firing, and reduced nociceptive traffic to primary afferent synaptic terminals, these findings suggest a mechanism that results in vHCA8WT-driven antinociception and analgesia.

### vHCA8 prolongs AHP via activation of Kv7 voltage-gated potassium channels

We further tested the hypothesis that vHCA8 prolongs AHP via activation of Kv7 channels (apparently due to altered calcium signaling). It is known that increased CA8 expression leads to decreased ER calcium release (Zhuang et al., 2015; Fu et al., 2017; Levitt et al., 2017; Zhuang et al., 2018; Upadhyay et al., 2019), resulting in lower cytosolic free calcium concentration. The Kv7 channels are the only potassium channels activated by low cytosolic calcium concentrations, in contrast to all other potassium channels involved in mediating AHP, which need higher calcium for activation. Then, Kv7 channel activation prolongs AHP.

**Figure 4:**
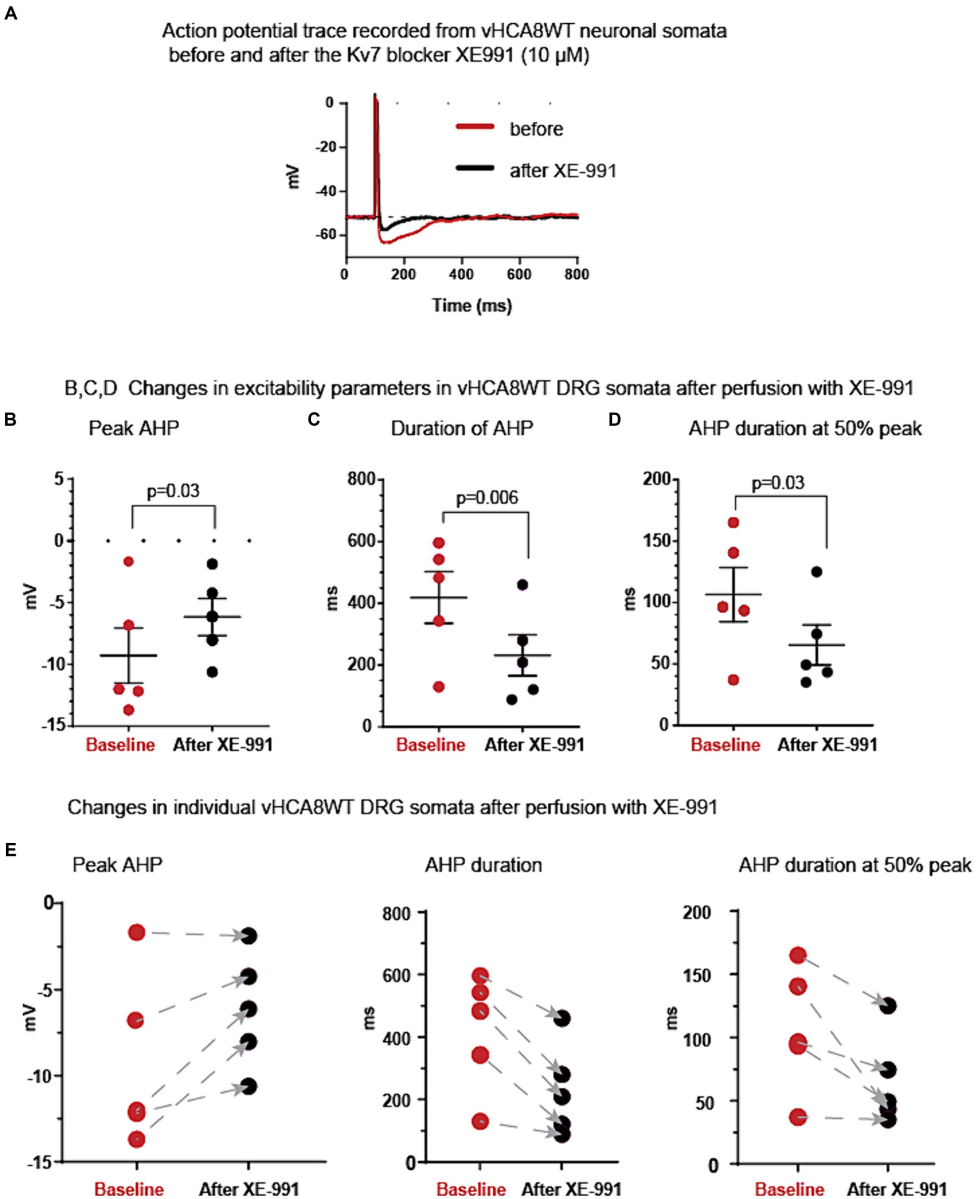
Kv7 selective inhibitor XE-991 reverses vHCA8WT prolonged AHP. **(A)** Representative AP traces recorded from vHCA8WT infected DRG neuronal somata before (red trace) and after XE-991 (10 μM) administration by perfusion in external bath solution (black traces). **(B)** XE-9991 resulted in a decrease of the peak amplitude of the AHP (black circles) compared to the baseline (red circles) (*p* = 0.03). Similarly, XE-991 administration **(C)** shortened the duration of AHP at the level corresponding to the resting membrane potential (*p* = 0.006); XE-991 also shortened the duration of AHP at the level corresponding to 50% peak AHP amplitude height **(D)** (*p* = 0.03). Means ± SEM are shown in the upper graph series. Horizontal bars above groups indicate statistically significant differences between groups. Decreases of the AHP parameters in individual somata after XE-991 perfusion are shown by arrows in lower graph series **(E)**.

To test whether the activation of Kv7 was involved in AHP prolongation after vHCA8WT treatment, we obtained recordings before and after perfusion of the Kv7-specific inhibitor XE-991 onto vHCA8WT-infected small DRG somata. Original representative action potential traces recorded from vHCA8WT-infected neurons before (red trace) and after (black trace) perfusion with XE-991 (10 μM) are shown in Figure 4A. As shown, XE-991 administration resulted in a decrease of the AHP peak amplitude, in shortening of the duration of the AHP, and the AHP duration at 50% peak (black circles) versus baseline (red circles), (Figures 4B–D, respectively).

This AHP reversal occurs consistently in all individual neurons, as shown by the direction of changes from pre- to post-perfusion with XE-991 in the lower graphs in Figure 4E.

Table 2 provides detailed EP parameters in vHCA8WT infected small neuronal DRG somata before (baseline) and after XE-991 perfusion (10 μM). XE-991 administration resulted in a significant decrease of peak AHP amplitude (−9.3 ± 4.9 mV vs. −6.2 ± 3.4 mV, *p* = 0.03), of the duration of AHP (419 ± 187 ms vs. 232 ± 148 msec, *p* = 0.006), and the duration of AHP at 50% amplitude (107 ± 49 ms vs. 65 ± 36 ms, *p* = 0.03) respectively. No other changes were observed.

**Table 2:** Comparison of electrophysiological parameters at baseline (untreated) and after the specific Kv7 inhibitor XE-991 in vHCA8WT-Infected small DRG neurons.

	**vHCA8WT** **(*n* = 5)**	**After XE-991 (10 μM)**	***p*-value**
Resting membrane potential (mV)	−50.1 ± 5.4	−51.3 ± 6.5	0.55
Peak action potential amplitude (mV)	137.4 ± 31.6	139.1 ± 31.7	0.91
Duration of action potential (ms)	14.3 ± 7.8	17.0 ± 5.3	0.13
Duration of action potential at 50% amplitude (ms)	4.4 ± 1.3	4.8 ± 1.5	0.51
Peak afterhyperpolarization amplitude (mV)	−9.3 ± 4.9	−6.2 ± 3.4	0.03
Duration of afterhyperpolarization (ms)	418.9 ± 187.0	231.7 ± 148.1	0.006
Duration of afterhyperpolarization at 50% amplitude (ms)	106.5 ± 49.2	65.4 ± 36.4	0.03

Comparison of electrophysiological parameters in a subset of vHCA8WT infected small-sized neuronal somata before and after perfusion with the selective Kv7 channel inhibitor XE-991 (10 μM). In contrast to controls, where no changes were observed (Supplementary Table S1), vHCA8WT-infected somata show evidence of upregulated M-currents which drive the AHP parameters (to higher values) and which are inhibited by treatment with the selective Kv7 channel inhibitor XE-991. Perfusion with XE-991 reverses the prolongation of AHP observed in vHCA8WT, to the point that AHP values after XE-991 were not different than those in Controls (Supplementary Table S2). Means ± SDs are shown. Comparisons were carried out using paired Student’s *t*-tests testing the hypothesis that administration of XE-991 would reverse the enhancement of duration and amplitude of AHP in vHCA8WT.

In contrast to the significant decrease in AHP parameters in vHCA8-infected DRG neurons, XE-991 did not reverse any AHP parameters in control cells (Supplementary Table S1), indicating the increased activity of Kv7 channels in the vHCA8 but not in the control group.

Furthermore, the values of the reversed AHP parameters by XE-991 in vHCA8-infected neurons did not differ significantly from those in controls, indicating a reversal back to a baseline status (Supplementary Table S2). Supplementary Table S2 shows AHP parameters in these neurons, which were infected with vHCA8WT and after being treated with XE-991, versus control neurons. These data show that there are no significant differences in AHP parameters between vHCA8WT-infected neurons treated with XE-991 and controls. Furthermore, a further comparison of AHP parameters after administration of XE-991 (10 μM) in uninfected DRG neuronal somata (Supplementary Table S1) showed no change in AHP parameters with the Kv7 channel inhibitor. These data indicate that the selective upregulation of the Kv7 M-current occurs in vHCA8WT-infected neurons only but not in controls, presumably because of CA8-mediated reduction of intracellular calcium levels and Kv7 channel activation in the former.

A few other potassium channels have been implicated in controlling AHP in DRG neurons, and although those are mainly activated by higher cytosolic calcium (Sarantopoulos et al., 2007), we additionally tested if those might have also affected AHP. In contrast to the selective Kv7 blocker, XE-991, which diminished the AHP parameters (Figure 4 and Table 2), blockers that are selective for other potassium channels failed to reduce AHP parameters. Specifically, the selective blocker for the ATP -sensitive potassium (K_ATP_) channels, glibenclamide (Supplementary Table S3A and Supplementary Figure S2A), the selective blocker for the calcium-activated large conductance potassium (BK) channels, iberiotoxin (Supplementary Table S3B and Supplementary Figure S2B), and the selective blocker for the calcium-activated small conductance potassium (SK) channels, apamin (Supplementary Table S3C and Supplementary Figure S2C) failed to reduce AHP parameters, ruling out these other channels as mediators of the prolonged AHP. Prolonging the AHP is mediated predominantly via Kv7 channels, further supporting our hypothesis.

### Activity suggestive of active Kv7 channels is present in vHCA8-infected neurons but not in controls

Current clamp recordings in Tyrode’s solution again exhibited prolonged AHP durations in vHCA8WT (262 ± 83.6 ms) vs. controls (101.7 ± 84.8 ms) (*p* = 0.005), consistent with the previous experiments. To further confirm that this was due to the activation of Kv7 channels that convey M-current, after the current-clamp recordings, we proceeded to acquire voltage-clamp recordings from vHCA8 infected small DRG neurons and from controls which were non infected small neurons (Figure 2). Currents were recorded as per previously published protocol (Passmore et al., 2003; Zheng et al., 2013) by holding the membrane potential at -20 mV and applying a square hyperpolarizing pulse at −50 mV for 1 s and then back to −20 mV, at baseline and after perfusion of 10 μM XE-991 in the bath. Since Kv7 is a non-inactivating current, this protocol minimized contributions from other inactivating currents, and the selective blocker XE-991 ensured additional selectivity. XE-991 resulted in statistically significant current inhibition only in DRG neuronal somata infected with vHCA8WT (Figure 2A) but not in controls (Figure 2B). Our results indicate the presence of upregulated Kv7 currents in vHCA8-infected neuronal somata but not in controls.

Our results confirm that nociceptor treatment by vHCA8WT but not vHCA8MT results in selective activation of Kv7 voltage-gated potassium channels, prolonging AHP and enhancing refractoriness, thus reducing the frequency of action potential firing and neuronal excitability and the subsequent propagation of afferent nociceptive signals (Passmore et al., 2003). This reduction in neuronal excitability resulting from prolonged AHP likely drives analgesia and anti-hyperalgesia observed in published studies via suppressed nociceptive afferent traffic.

## Discussion

Most patients suffering from chronic pain, which is a prevalent and disabling condition, are inadequately treated, making the development of better analgesics a high priority. In the absence of alternatives, long-term opioid use in treating chronic pain has increased dramatically over the past few decades, and unwanted opioid effects, including dependence and abuse, are major public health concerns (Compton and Volkow, 2006; Paulozzi and Xi, 2008; Boudreau et al., 2009; Edlund et al., 2010a,b). In this context, the advantages of the JDNI8 rdHSV for the delivery of analgesic transgenes to nociceptors that have the potential to mediate antinociception and address this great unmet need for novel non-opioid analgesics.

Herein, we exploit this novel rdHSV-based gene delivery system that takes advantage of the natural tropism of HSV for sensory nerves. Establishing a molecular mechanism of Kv7 channel activation for vHCA8-induced analgesia and anti-hyperalgesia lends further support to the potential clinical-translational value of these findings for treating human chronic noncancer pain, given the established effectiveness of therapeutics based on this mechanism in prior human studies.

Our previous studies have reported a novel analgesic effect of CA8. In the current study, we have identified the corresponding underlying mechanism of this analgesia. We have found that vHCA8WT decreases nociceptor excitability, which has the potential to result into reduced trafficking of nociceptive signals to the CNS and putative analgesia. In particular, vHCA8 decreases primary afferent excitability via activated Kv7 potassium channels (Figures 2, 4). These channels provide a mechanistic link between CA8-based reduction of cytosolic free calcium, conferred by vHCA8WT gene therapy, and exogenous CA8 expression, resulting in downstream attenuation of primary afferent neuronal excitability. This conclusion is based on the following supporting evidence: (Fu et al., 2017) Our whole-cell current-clamp recordings show that infection of small-sized DRG neurons (corresponding to nociceptors) (Lawson, 2002) with vHCA8WT but not vHCA8MT causes prolongation of AHP (Figure 3 and Table 1; Levitt et al., 2017) This AHP prolongation is completely reversed by the Kv7-specific inhibitor XE-991 (Figure 4, Table 2, and Supplementary Table S2) but not impacted by other potassium channel inhibitors (Supplementary Figure S2 and Supplementary Table S3) (Upadhyay et al., 2019). This highlights the role of Kv7 as in mediating the prolongation of AHP in this setting (Berridge, 1993). Furthermore, the reversal of active upregulated Kv7 I_M_-currents by XE-991 in vHCA8WT-infected nociceptors but not in controls is demonstrated by our voltage-clamp recording.

Transmembrane Kv7.2, Kv7.3, and K_V_7.5 channel proteins (encoded by KCNQ genes) are widely expressed as tissue-specific hetero-tetramers in nociceptors (Wu et al., 2017), wherein they mediate I_M_ currents resulting in inhibitory effects. Primary afferent nociceptors maintain experimental chronic pain in rodent models, and this strongly supports our targeting primary afferent nociceptors with localized vHCA8WT gene therapy, which produces Kv7 activation (Okun et al., 2012) to suppress of neuronal excitability.

While other potassium channels involved in attenuating neuronal excitability, such as the calcium-activated potassium channels (Sarantopoulos et al., 2007) or the ATP-sensitive potassium channels (Kawano et al., 2009), are activated by elevated cytosolic calcium, this is not the case with Kv7 channels. Kv7 voltage-gated potassium channels are instead activated by lower cytosolic calcium, such as after suppression by CA8, thus resulting in upregulated I_M_ (M-currents) (Marrion et al., 1991; Selyanko and Brown, 1996a; Selyanko and Brown, 1996b). M-currents have been shown to exert powerful control on neuronal excitability (Brown and Passmore, 2009; Wang and Li, 2016), and even small reductions in the I_M_ from pharmacological inhibition, physiological modulation, or mutation can result in dramatic increases in neuronal excitability (Peters et al., 2005; Luebke and Chang, 2007) and pain resilience (Yuan et al., 2021). I_M_ are also involved in controlling the AHP, serving as a major regulator of neuronal excitability (Luebke and Chang, 2007; Sanchez et al., 2011; Greene and Hoshi, 2017), as well as the frequency at which neurons fire while receiving continuous excitatory input (Madison and Nicoll, 1984; Greene and Hoshi, 2017). It is further well-established that all DRG neurons express Kv7 immunoreactivity and I_M_ currents (Passmore et al., 2003), which are not significantly altered by inflammation (Cisneros et al., 2015).

Our findings show that the prolongation of the AHP by activation of Kv7/I_M_ currents is consistent with other published studies (Gu et al., 2005; Peters et al., 2005; Greene and Hoshi, 2017) and mechanistically links Kv7 channel activation to decreased intracellular calcium ([Ca^2+^]_i_) (Gamper and Shapiro, 2003). Upregulated I_M_ results in suppression of nociceptor excitability, which leads to antinociception. This mechanism is clinically pertinent because of its translational capacity to provide significant non-opioid analgesia. By vHCA8WT enhancing AHP, I_M_ hyperpolarizes the membrane, makes it refractory to excitation and to the generation of spikes, and reduces the release of excitatory neurotransmitters at presynaptic terminals, thus resulting in the exploitation of a natural mechanism to decrease transmission of pain signaling.

Three phases of AHP contribute to refractoriness: fast AHP (fAHP) lasting 2–5 ms, medium AHP (mAHP) ranging between 100 to 300 ms, and slow AHP (sAHP) > 1 s to 2 s (Madison and Nicoll, 1984; Storm, 1990). Considering their time course, the changes we have observed likely correspond to mAHP, a phase that has been shown to depend on Kv7 channels. Yet, other channels have been suggested as potential mediators of mAHP, such as SK calcium-activated potassium channels and HCN channels conveying I_h_ current (Storm, 1989; Stocker et al., 1999). However, there is variability, and distinct channels are responsible for mAHP depending on specific neuronal types and on membrane potentials (Gu et al., 2005). Our findings highlight the Kv7/I_M_ as a dominant contributor to mAHP since SK activation requiring higher [Ca^2+^]_i_ for activation, and it was not surprising that apamin failed to reverse vHCA8WT-induced prolongation of the AHP. In contrast, HCN channels are activated at a much more hyperpolarized potential range (around –80 mV) (Gu et al., 2005) and not in the −60 to −50 mV range recorded for our neurons. Furthermore, HCN/I_h_ currents are activated by higher [Ca^2+^]_i_, too, released via IP_3_ receptors (Neymotin et al., 2016), which is not the case in our vHCA8-infected cells.

Our findings are also consistent with those by Peters et al. (2005), who reported that 10 μM XE991 reduces mAHP amplitude by ∼75% after train-of-four spikes and that this was mediated via I_M_ but not via any apamin-sensitive SK currents. Gu et al. have also reported that at membrane potentials around −60 mV, mAHP is generated mainly by Kv7/I_M_, with little or no contribution from apamin-sensitive SK channels, whereas at more hyperpolarized membrane potentials (∼ −80 mV), the I_h_ becomes the main contributor to mAHP. I_h_ is deactivated at around −60 mV and thus unlikely to contribute to AHP at this range (Storm, 1989).

Kv7/I_M_ is reported as the major contributor to sAHP (Greene and Hoshi, 2017), but we believe that our findings are more consistent with the mAHP. This is true when taking into consideration the time course, as well as sAHP is active at membrane potentials that are more negative than those at which I_M_ activity is typically observed in our cells (Greene and Hoshi, 2017), and since sAHP is calcium-dependent.

Though the identity of Kv7 was identified pharmacologically, XE-991 is recognized as a highly selective antagonist for Kv7 (Wang et al., 1998, 2000; Gu et al., 2005; Greene et al., 2017), and others have also reported on the suppression of mAHP mainly by M-channel blockade by XE-991 (Storm, 1989; Gu et al., 2005).

Kv7/I_M_ currents have a threshold for activation that is usually higher than the typical neuronal resting potentials, with greater activity upon depolarization. Because of this and its lack of inactivation, these currents have a major impact on neuronal excitability (Hernandez et al., 2008). Kv7/I_M_ currents have slow kinetics and traditionally have been considered as unlikely to be significantly induced by a single action potential. To account for this in our protocol, we selected for analysis the AP-AHP traces elicited by the fifth in a sequence of five AP complexes, elicited by a supramaximal 2.5 nA current pulse to ensure spike firing. On the other hand, it should also be noted that even a single action potential can elicit Kv7/I_M_ activity to affect AHP (Madison and Nicoll, 1984; Gu et al., 2005), and also in a fashion that does not require high [Ca^2+^]_i_ (thus consistent with I_M_) (Storm, 1989).

In fact, although there is a relative lack of data on native I_M_ kinetics in mammalian neurons at depolarized potentials above −20 mV (Wang et al., 1998; Gu et al., 2005), studies have confirmed that even single depolarizing AP spikes can generate substantial I_M_ currents. Gu et al. have reported explicitly that I_M_ currents are activated significantly during a single AP that lasts only 1–2 ms, that this can generate quite prominent mAHPs even after a single AP spike (Gu et al., 2005), and that I_M_ blockade attenuates the mAHP at −60 mV. They have also specifically reported that, due to their slow kinetics, the Kv7 channels open late by a single AP, with I_M_ increases up to 30–40% of their full open probability and of their full I_M_-conductance by a single AP. However, this 30–40% fraction of full conductance is sufficient to account for the observed single-AP mAHP duration at about −60 mV (Gu et al., 2005), and this also agrees with our findings.

Other potassium channels may be inhibited to some extent by XE-991 as well, such as the Kv1.2 (Zhong et al., 2010), but at a much higher IC_50_ (>100 μM) versus 0.98 μM for I_M_. Furthermore, any contribution of Kv1.2 in our studies is extremely low. Kv1.2 channels are mainly present in motor neurons, while in DRG neurons these channels are primarily expressed in larger-diameter neurons (larger myelinated fibers) (Rasband et al., 2001; Fan et al., 2014) but not in small neurons, as studied in our experiments. Additionally, these channels in neurons are mainly involved in contributing to the resting membrane potential and mediating the repolarization of action potentials (Baronas et al., 2018; Zhang et al., 2021) but not AHP.

Neuronal mAHP was shown to be primarily mediated by I_M_ (at depolarized potentials) and I_h_ (at more negative potentials), with little or no contribution by SK channels (Storm, 1989; Gu et al., 2005) So, our results are in agreement with those of Gu et al., who showed that specific I_M_ blocker XE-991 (10 μM) suppressed the mAHP following even one AP evoked by current injection at −60 mV. The HCN/I_h_ blocker ZD7288 (*4-ethylphenylamino-1,2-dimethyl-6-methylaminopyrimidinium chloride*; 10 μM) fully suppressed the mAHP at −80 mV, but had little effect at −60 mV, whereas XE-991 did not measurably affect the mAHP at −80 mV (Storm, 1989; Gu et al., 2005). So, although we did not block I_h_, we think that this current is unlikely to have affected the AHP in our setting, which is also at the −60 mV range wherein these neurons operate. Gu et al. also found that blockade of calcium-activated potassium channels of the SK type by apamin (100–400 nm) failed to affect the mAHP, also in agreement with our results.

In our studies, the contribution of large conductance BK channels is likely low since they contribute to fast AHP. As for the small conductance SK channels, their contribution to medium AHP is either minimal or none (Storm, 1989; Gu et al., 2005). Contribution by the HCN/I_h_ current to medium AHP in our setting of −60 mV is also considered to be minimal. Previous studies have shown that HCN/I_h_ blocker ZD7288 (*4-ethylphenylamino-1,2-dimethyl-6-methylaminopyrimidinium chloride*; 10 μM) fully suppressed the mAHP at −80 mV, but had little effect at −60 mV, whereas XE-991 did not measurably affect the mAHP at −80 mV (Storm, 1989; Gu et al., 2005). Although, Kir6 containing K_ATP_ channels have been also implicated in controlling the AHP (Tanner et al., 2011), we do not think that these may have contributed to the differences that we observed because they are also activated by higher [Ca_2+_]_i_ concentrations (Kawano et al., 2009) (contrary to our proposed mechanism) and their specific blocker glibenclamide had no effects.

Kv7 channels have been implicated in controlling the RMP, but this effect has been reported only on axons, on the nodes of Ranvier, and on axon initial segment (AIS) wherein they are highly concentrated, as well as on axon terminals (Devaux et al., 2004; Greene and Hoshi, 2017). However, no significant changes in the RMP were observed in our findings. This is likely because our recordings involved neuronal somata, and not axons or AIS.

It seems that during a single AP, (especially after four preceding AP spikes), 30 to 40% of I_M_ full conductance would be enough to prolong AHP but would not hyperpolarize the RMP. Furthermore, while Kv7.2 control RMP only in axons, it is unclear whether they also do the same in somata but is seems in the later location are more involved in attenuating afterdepolarization responses, rather than RMP (Hu et al., 2007). Interestingly, and in support of our findings, Hu et al., have reported that the application of retigabine or XE-991 to neuronal somata had no significant effect on the RMP. Peters et al. (2005) also reported no changes in RMP at baseline conditions between mutants and controls, and no changes in AP parameters, using experiments in neurons of transgenic mice that conditionally express dominant-negative KCNQ2 subunits, suggestive of no contribution of M-current to RMPs, but they attributed changes in excitability to AHP and spike-frequency adaptation.

Despite our showing the potential of carbonic anhydrase expression to attenuate excitability via Kv7 channels, a few limitations need to be discussed further. Carbonic anhydrase isoforms mediate diverse intracellular and extracellular signaling effects, by causing pH shifts, pH-mediated modulation of voltage- and ligand-gated ion channels and gap junctions, which also affect neuronal excitability (Ruusuvuori and Kaila, 2014). Therefore, in addition to the effects via Kv7 activation, which we clearly demonstrated, any pH-dependent contributions to altered excitability cannot be ruled out in our study, and this may be a limitation.

Furthermore, we acknowledge that we know neither which subtypes of sensory neurons are preferably transduced with HSV in this context, nor their specific Kv7 expression, and this is another limitation, but introduces an interesting question that remains to be further studied. Despite analogies between rodent and human DRG, extrapolating these results to humans may be another interesting question that remains to be further studied.

Using human DRGs for similar electrophysiological experiments is an exciting concept and would add great value, as well as studying and further characterizing the responses of different sensory neuronal populations and subtypes regarding their specific Kv7 expression in parallel with their preferential transduction with HSV, considering that peptidergic nociceptors may be preferentially transduced by HSV (Herbort et al., 1989).

Though Kv7 is a highly relevant molecular target for chronic pain therapy, there are no Kv7 activators currently available for clinical use (Konishi et al., 2018; Perdigoto et al., 2018). Thus, our proposed rdHSV-based vHCA8WT gene therapy, which results in downstream activation of Kv7 and decreased primary afferent excitability, may have the potential to possibly provide a reasonable, more efficacious, and potentially safer localized target-specific alternative to opioids for clinical analgesia and anti-hyperalgesia. This reduced hyperexcitability of targeted somatosensory neurons, is subsequent to the delivery of the CA8 transgene specifically to their nuclei, by localized application of this disease-free rdHSV. Additionally, this localized delivery of this novel Kv7 activator is likely to avoid the off-target effects and other safety concerns seen with the systemic administration of prior Kv7 activators used to successfully treat various forms of chronic pain. This novel mechanism has the potential to translate in future pharmacological applications that can address the major unmet need for non-opioid analgesics for the treatment of chronic pain.

## Data availability statement

The original contribution presented in the study are included in the article/Supplementary material, further inquires can be directed to the corresponding author’s.

## Ethics statement

The animal study was approved by University of Miami Institutional Animal Use and Care Committee (IACUC). The study was conducted in accordance with the local legislation and institutional requirements.

## Author contributions

MK: Data curation, Formal analysis, Methodology, Validation, Writing – review & editing. GZ: Project administration, Supervision, Writing – review & editing. WG: Conceptualization, Investigation, Methodology, Resources, Supervision, Validation, Writing – review & editing. MM: Data curation, Formal analysis, Investigation, Methodology, Writing – original draft, Writing – review & editing. MZ: Data curation, Formal analysis, Investigation, Methodology, Writing – original draft, Writing – review & editing. JG: Conceptualization, Methodology, Project administration, Validation, Writing – review & editing. YK: Data curation, Formal analysis, Writing – review & editing. AL: Writing – original draft, Writing – review & editing. W-MK: Methodology, Writing – review & editing. RL: Conceptualization, Funding acquisition, Investigation, Methodology, Project administration, Resources, Supervision, Validation, Visualization, Writing – original draft, Writing – review & editing, Data curation, Formal analysis, Software. KS: Formal analysis, Investigation, Methodology, Project administration, Resources, Software, Supervision, Validation, Visualization, Writing – original draft, Writing – review & editing, Conceptualization, Data curation.
