# Supplementary material for: rdHSV-CA8 non-opioid analgesic gene therapy decreases somatosensory neuronal excitability by activating Kv7 voltage-gated potassium channels
Source: Front Mol Neurosci. 2024 May 9;17:1398839. doi: 10.3389/fnmol.2024.1398839 (PMC11112096; doi:10.3389/fnmol.2024.1398839)
Supplement: Supplementary file 1 [file Data_Sheet_1.docx]

**Supplementary Material**

**Supplementary Table 1. Comparison of electrophysiological parameters in control small DRG neurons before and after the Kv7 inhibitor XE-991 (10 µM).** Small-sized control DRG neuronal somata infected with vHCA8MT, or uninfected (vehicle), exhibited no differences in AHP parameters after blocking Kv7 by XE-991 as compared to baseline. This is in contrast to vHCA8WT-infected neurons wherein XE-991 shortened the AHP (Fig. 3; Table 2). Means±SDs are shown. Comparisons were carried out with paired Student’s t-tests testing the hypothesis that administration of XE-991 would reduce the amplitude and the duration of AHP compared to the baseline values.

**Supplementary Table 1.**

|  | **Baseline**  **(n=6)** | **After XE-991 (10µM)** | **P-Value** |
| --- | --- | --- | --- |
| Resting membrane potential (mV) | -53.32±3.45 | -52.65±4.79 | 0.54 |
| Peak action potential amplitude (mV) | 140.75±21.24 | 140.38±19.96 | 0.95 |
| Duration of action potential (ms) | 5.08±1.15 | 5.73±1.71 | 0.39 |
| Duration of action potential at 50% amplitude (ms) | 2.65±0.20 | 2.58±0.20 | 0.17 |
| Peak afterhyperpolarization amplitude (mV) | -10.31±4.48 | -10.74±6.52 | 0.69 |
| Duration of afterhyperpolarization (ms) | 68.21±49.37 | 71.6±43.71 | 0.37 |
| Duration of afterhyperpolarization at 50% amplitude (ms) | 17.26±6.7 | 17.75±5.69 | 0.53 |

**Supplementary Table 2.** **Comparison of AHP peak, AHP duration, and AHP duration at 50% amplitude between controls and vHCA8WT-Infected somata after administration of the selective Kv7 channels inhibitor XE-991.** The values of the AHP parameters after the administration of XE-991 did not differ from those values in controls. Means ± SDs are shown. Comparisons were carried out with unpaired Student’s t-tests.

**Supplementary Table 2.**

|  | **Controls (vHCA8MT and uninfected cells)**  **(n=9)** | **vHCA8WT after XE-991 (10µM)**  **(n=5)** | **P-Value** |
| --- | --- | --- | --- |
| Peak afterhyperpolarization amplitude (mV) | -6.5±2.3 | -6.2±3.4 | 0.430 |
| Duration of afterhyperpolarization (ms) | 114.0±71.67 | 231.7±148.1 | 0.15 |
| Duration of afterhyperpolarization at 50% amplitude (ms) | 31.3±21.3 | 65.4±36.4 | 0.11 |

**Supplementary Table 3.A. Comparison of electrophysiological parameters before (at baseline) and after the K_ATP_ channel inhibitor glibenclamide in vHCA8WT-infected small DRG neurons.** Small-sized DRG neuronal somata infected with vHCA8WT treated with K_ATP_ channel inhibitor glibenclamide (10 µM) exhibited no differences in AHP parameters as compared to neuronal somata at baseline. In contrast to the specific Kv7.2 inhibitor XE-991 (Shown in Figure 3 and Table 2), the peak AHP amplitude and duration did not change by glibenclamide, indicating that the vHCA8WT enhancing effect on AHP is not mediated via K_ATP_ channels. Means±SDs are shown. Comparisons were carried out with paired Student’s t-tests testing the hypothesis that administration of glibenclamide would reduce the amplitude and the duration of AHP compared to the baseline values.

**Supplementary Table 3.A.**

|  | **Baseline**  **(n=4)** | **After Glibenclamide (10µM)** | **P-Value** |
| --- | --- | --- | --- |
| Resting membrane potential (mV) | -57.3±9.7 | -60.4±9.9 | 0.4 |
| Peak action potential amplitude (mV) | 140.3± 16.3 | 144.2±17.8 | 0.09 |
| Duration of action potential (ms) | 6.9±1.4 | 7.1±0.9 | 0.42 |
| Duration of action potential at 50% amplitude (ms) | 2.7±0.3 | 2.7±0.2 | 0.6 |
| Peak afterhyperpolarization amplitude (mV) | -8.2±2.7 | -7.8±2.5 | 0.48 |
| Duration of afterhyperpolarization (ms) | 363.3±209.4 | 423.3±253.8 | 0.24 |
| Duration of afterhyperpolarization at 50% amplitude (ms) | 69.1± 17.7 | 66.2±11.9 | 0.49 |

**Supplementary Table 3.B. Comparison of electrophysiological parameters before (at baseline) and after the BK KCa channel inhibitor iberiotoxin (100 nM) in vHCA8WT-infected small DRG neurons.** Comparison of electrophysiological parameters in six vHCA8WT infected small-sized neuronal somata before and after bath perfusion with the selective large-conductance (BK) calcium-activated potassium (KCa) channel inhibitor Iberiotoxin (100nM). In contrast to the specific Kv7.2 inhibitor XE-991 (Shown in Figure 3 and Table 2), the peak AHP amplitude and duration did not change by iberiotoxin, indicating that the vHCA8WT enhancing effect on AHP is not mediated via BK channels. Means±SDs are shown. Comparisons were carried out with paired Student’s t-tests testing the hypothesis that administration of iberiotoxin would reduce the parameters describing the duration and amplitude of AHP from the baseline values.

**Supplementary Table 3.B.**

|  | **Baseline**  **(n=6)** | **After Iberiotoxin**  **(100 nM)** | **P-Value** |
| --- | --- | --- | --- |
| Resting membrane potential (mV) | -61.1±6.5 | -63.5±5.5 | 0.01 |
| Peak action potential amplitude (mV) | 153.2±20.8 | 151.0±16.1 | 0.41 |
| Duration of action potential (ms) | 7.8±2.1 | 6.6±1.6 | 0.005 |
| Duration of action potential at 50% amplitude (ms) | 2.9±0.5 | 2.7±0.5 | 0.048 |
| Peak afterhyperpolarization amplitude (mV) | -10.2±1.4 | -11.9±2.0 | 0.045 |
| Duration of afterhyperpolarization (ms) | 363.2±173.4 | 364.5±168.6 | 0.87 |
| Duration of afterhyperpolarization at 50% amplitude (ms) | 84.8±46.0 | 91.3±40.4 | 0.24 |

**Supplementary Table 3.C. Comparison of electrophysiological parameters before (at baseline) and after the specific SK KCa channel inhibitor apamin (1 µM) in vHCA8WT-infected small DRG neurons.** Comparison of electrophysiological parameters in four vHCA8WT infected small-sized neuronal somata before and after bath perfusion with the selective small-conductance (SK) calcium-activated potassium (KCa) channel inhibitor apamin (1µM). In contrast to the specific Kv7.2 inhibitor XE-991 (Shown in Figure 3 and Table 2), the peak AHP amplitude and duration did not change by apamin, indicating that the vHCA8WT enhancing effect on AHP is not mediated via SK channels. Means±SDs are shown. Comparisons were carried out with paired Student’s t-tests testing the hypothesis that administration of apamin would reduce the parameters describing the duration and amplitude of AHP from the baseline values.

**Supplementary Table 3.C.**

|  | **Baseline**  **(n=4)** | **After Apamin**  **(1 µM)** | **P-Value** |
| --- | --- | --- | --- |
| Resting membrane potential (mV) | -59.8±5.7 | -61.0±6.4 | 0.33 |
| Peak action potential amplitude (mV) | 164.9±41.5 | 163.6±31.5 | 0.84 |
| Duration of action potential (ms) | 7.5±1.3 | 6.9±1.0 | 0.03 |
| Duration of action potential at 50% amplitude (ms) | 2.8±0.3 | 2.7±0.1 | 0.14 |
| Peak afterhyperpolarization amplitude (mV) | -10.0±1.6 | -11.2±1.5 | 0.1 |
| Duration of afterhyperpolarization (ms) | 285.8±37.0 | 306.8±23.1 | 0.1 |
| Duration of afterhyperpolarization at 50% amplitude (ms) | 74.7±26.8 | 85.3±26.5 | 0.14 |

**Supplementary Figures**

**Supplementary Figure 1.**


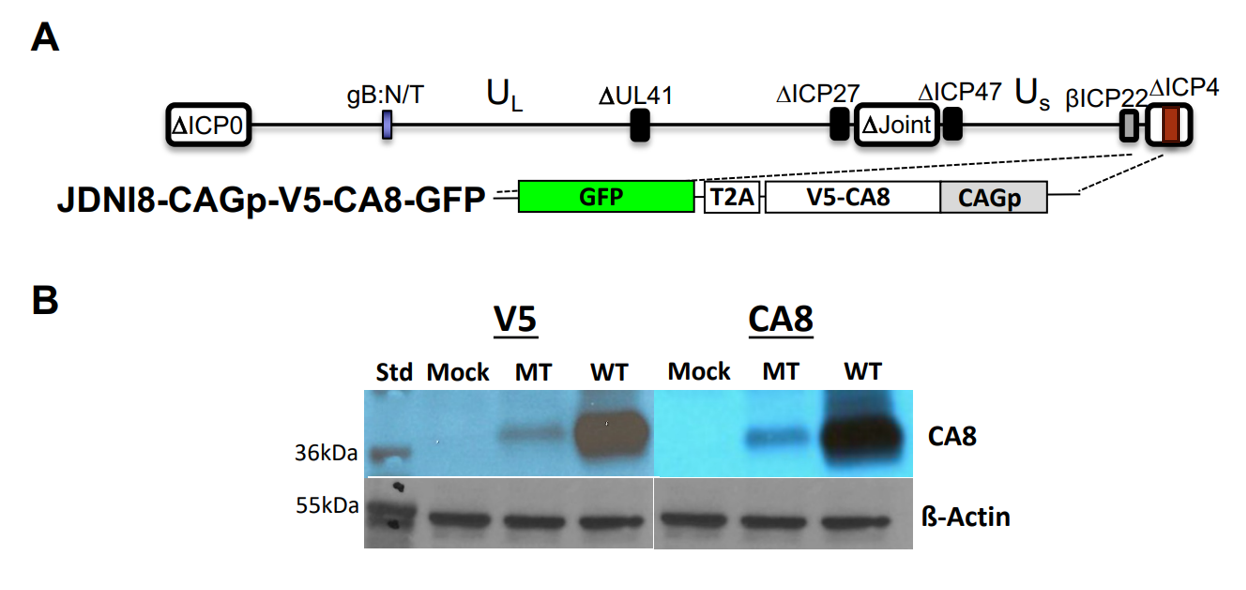


**Supplementary Figure 2.**

**Supplementary Figure Captions:**

**Supplementary Figure 1. Schematic Diagram of HSV HCA8 Constructs and Expression in Primary Rat DRG Neurons *In Vitro*. A.** The JDNI8 vector was derived from JDNI5 (36, 37). The BAC elements are located between *lox*P sites in the UL37-UL38 intergenic region (82). U_L_, unique long segment; Us, unique short segment. Deletions in JDNI8 are indicated by black boxes. JDNI8 contains the entry-enhancing D285N/A549T(N/T) mutations in the gB gene (gB: NT) (83), and a CAG (CAGp)-CA8*-V5-T2A-eGFP cassette (green) in the ICP4 site (red). The blue rectangle box (βICP22) indicates the conversion of the ICP22 IE gene to early-expression kinetics by deletion of promoter TAATGARAT. **B.** Western blots were processed for SDS-PAGE, transferred to PVDF membranes, and immunoblotted with antibodies to CA8 and the V5 tag using ß-actin as a loading control for primary rat DRG neuronal cell lysates2 days after infection with vHCA8WT or vHCA8MT (MOI=5). The correct-sized band was identified in each case. vHCA8MTvirus was barely detectable above the background as compared to vHCA8WT virus.

**Supplementary Figure 2. The Effect of Other Potassium Channel Inhibitors on the AHP Peak Amplitude, AHP Duration and AHP Duration at 50% Amplitude. A.** Electrophysiologic recordings from vHCA8WT infected DRG neuronal somata before (red circles) and after the administration of the selective K_ATP_ channel blocker glibenclamide (10 µM) by perfusion in external bath solution (black circles). Glibenclamide failed to decrease the AHP peak amplitude (left panel), failed to shorten the AHP duration (middle and right panels) (black circles) compared to baseline (red circles) and failed to shorten the AHP duration at 50% peak (right panel). **B.** Electrophysiologic recordings from vHCA8WT infected DRG neuronal somata before (red circles) and after the administration of the selective BK channel iberiotoxin (100nM) by perfusion in external bath solution (black circles). Iberiotoxin failed to decrease the peak amplitude of the AHP (left panel),- failed to shorten the AHP duration (middle panel) (black circles) compared to baseline (black circles) and failed to shorten the AHP duration at 50% peak (right panel). **C**. Electrophysiologic recordings from vHCA8WT infected DRG neuronal somata before (red circles) and after the administration of the selective SK channel inhibitor apamin (1µM) by perfusion in external bath solution (black circles). Apamin failed to decrease the peak amplitude of the AHP (left panel), failed to shorten the AHP duration (middle panel) (black circles) compared to baseline (red circles) and failed to shorten the AHP duration at 50% peak (right panel). These results differ from those observed with Kv7 specific inhibitor XE-991, which reversed the changes in AHP parameters associated with vHCA8WT infection (Figure 3; Table 2), but not in controls (Suppl. Table 1). Means ± SEM are shown. Horizontal bars above groups indicate statistically significant differences between groups.

**Supplementary References:**

**82. Gierasch W W, Zimmerman DL, Ward SL, Vanheyningen TK, Romine JD, Leib DA. Construction and characterization of bacterial artificial chromosomes containing HSV-1 strains 17 and KOS. J Virol Methods. 2006;135(2):197-206.**

**83. Uchida H, Chan J, Goins WF, Grandi P, Kumagai I, Cohen JB, et al. A double mutation in glycoprotein gB compensates for ineffective gD-dependent initiation of herpes simplex virus type 1 infection. J Virol. 2010;84(23):12200-9.**
